# Toward a Brighter Future for Psychology as an Observation Oriented Science

**DOI:** 10.3390/bs2010001

**Published:** 2012-01-16

**Authors:** James W. Grice, Paul T. Barrett, Liz A. Schlimgen, Charles I. Abramson

**Affiliations:** 1 Department of Psychology, Oklahoma State University, 116 North Murray, Stillwater OK 74074, USA; E-Mails: liz.schlimgen@gmail.com (L.S.), charles.abramson@okstate.edu (C.A.); 2 The University of Auckland, Private Bag 92019, Auckland 1142, New Zealand; E-Mail: paul@pbarrett.net

**Keywords:** Observation Oriented Modeling, research methods, null hypothesis significance testing, integrated models

## Abstract

Serious criticisms of psychology’s research practices and data analysis methods date back to at least the mid-1900s after the Galtonian school of thought had thoroughly triumphed over the Wundtian school. In the wake of Bem’s (2011) recent, highly publicized study on psi phenomena in a prestigious journal, psychologists are again raising serious questions about their dominant research script. These concerns are echoed in the current paper, and Observation Oriented Modeling (OOM) is presented as an alternative approach toward data conceptualization and analysis for the social and life sciences. This approach is rooted in philosophical realism and an attitude toward data analysis centered around causality and common sense. Three example studies and accompanying data analyses are presented and discussed to demonstrate a number of OOM’s advantages over current researcher practices.

## 1. Introduction

Daryl Bem’s [1] recently published and highly publicized paper, *Feeling the Future: Experimental Evidence for Anomalous Retroactive Influences on Cognition and Affect*, has generated a great deal of discussion and controversy in the popular culture as well as within the scientific community. Interestingly, much of the controversy among psychologists and other scientists has not revolved around the topic of the article—psi phenomenon, or ESP—but rather around the research methodology employed in the reported studies. In every way Bem followed the established protocol for conducting psychological research, which LeBel and Peters [2] refer to as the Modal Research Practice (MRP). He recruited hundreds of research participants, used randomization routines to present stimuli, analyzed his data using null hypothesis significance testing, and reported nine different studies demonstrating the experimental effect. As Roberts [3] points out, it is only by following accepted research practice that Bem could have expected to publish his controversial results in a mainstream, competitive journal. Skeptics were of course quick to discuss the numerous problems with specific aspects of Bem’s research design and statistical analyses (e.g., see Alcock [4]), but every published study is understood to possess flaws and limitations. The central and disturbing question therefore became, how can the research methods of psychology, *prima facie*, appear more apt for pseudoscience than for genuine scientific discovery? LeBel and Peters argued that Bem’s studies essentially require a choice to be made between revising fundamental beliefs about time and causality or questioning the efficacy of the MRP. They chose the latter and addressed three primary areas of concern: (1) misuse of null hypothesis significance testing, (2) failure to appreciate the importance of exact replication, and (3) lack of attention to the details of measurement and to the subtleties of experimental manipulations. Roberts [3] echoed these criticisms and listed additional, specific concerns with the statistical reasoning and analysis methods used in Bem’s studies.

Many of the individual ingredients of psychology’s research and analysis paradigm have been criticized over the years, and hence this latest round of concerns is nothing new. Writing in 1967, for instance, David Bakan [5] launched an early salvo against the rationale underlying the *p*-value as well as its ubiquitous employment as the gatekeeper to meaningful (or at least publishable) results. Perhaps even more well known is Paul Meehl’s [6] famous statement that the universal reliance on null hypothesis significance testing is “...one of the worst things that ever happened in the history of psychology” (p. 817). The limitations and potentially obfuscating traps associated with aggregate statistical analyses, like those used by Bem, were debated as early as the 1950s after the “triumph of the aggregate” had occurred in psychological research (see Danziger [7]). Skinner [8] similarly recognized long ago the limitations of relying too heavily on large-sample, between-subjects experimental designs and mechanized statistical analysis for understanding behavior,

“In essence, I suddenly found myself face to face with the engineering problem of the animal trainer. When you have the responsibility of making absolutely sure that a given organism will engage in a given sort of behavior at a given time, you quickly grow impatient with theories of learning. Principles, hypotheses, theorems, satisfactory proof at the .05 level of significance that behavior at a choice point shows the effect of secondary reinforcement— nothing could be more irrelevant. No one goes to the circus to see the average dog jump through a hoop significantly oftener than untrained dogs raised under the same circumstances, or to see an elephant demonstrate a principle of behavior.” (p. 228).

The methodological and analysis critiques of yesterday are no less valid today; consequently, the question of how to cure the maladies of modern psychological research is still relevant. Lebel and Peters [2] offer as remedies a more judicious use of null hypothesis significance testing, holding exact replication in higher regard, and routine checks on the internal validity of measurement and experimental procedures. Roberts [3] calls for at least one exact replication accompanying each journal submission, open access to data, and a journal of exact replication. Wagenmakers *et al.* [9] state simply that psychologists must change the ways they analyze their data, essentially abandoning null hypothesis significance testing as the primary analysis tool. All of these suggestions are worthy of serious consideration, but they are not likely to have an impact on most areas of research unless they are accompanied by a fundamental shift in philosophy. From our perspective, most domains of psychology are wedded to positivist philosophy because of the MRP. As discussed by Grice [10], many of the elements of the MRP have their origin in positivist philosophy which in turn restricts the ways psychologists frame their research questions. Karl Pearson [11], for instance, regarded science as essentially “sensation sorting”, which is consistent with Bem sorting through a small mountain of responses gathered from numerous participants in order to statistically detect a weak signal to which he could apply a post hoc explanation. To break free from these restrictions, psychologists must be willing to adopt new methods and try new ways of thinking consistent with a fundamentally different philosophical outlook. 

Observation Oriented Modeling (OOM) was proposed by Grice [10] as one alternative avenue of thought. Consistent with the philosophy of moderate realism in the tradition of Aristotle and St. Thomas Aquinas, OOM provides a novel way of conceptualizing psychological research which may result in several distinct benefits. First, the methods associated with OOM eschew null hypothesis significance testing and related techniques of estimating abstract population parameters via a sampling scheme. These methods are, compared to most statistical analyses employed in the MRP, simple to use, flexible, and relatively free of assumptions. Second, consistent with Aristotle’s philosophy, OOM employs a richer view of causality and brings causal understanding to the forefront of psychological investigation. Lastly, related to the importance of causality, OOM challenges researchers to construct *integrated models* that represent the structures and processes that constitute the phenomena under investigation. These models can serve as effective counters to the “methodolatry” [5] and “statisticism” [12] plaguing the MRP. With three examples to follow, we demonstrate these potential benefits of OOM with the hope of charting a more productive course for psychological research.

## 2. Example 1: Simplified Analysis and the Eye Test

As an initial look at the Observation Oriented Modeling (OOM) approach toward data conceptualization and analysis, consider a simple study published by Buss and his colleagues [13]. Undergraduate participants imagined a past, current, or desired romantic partner and then chose one of two scenarios of infidelity they found most distressing: (a) imagining their partner having sexual intercourse with another person (sexual infidelity), or (b) imagining their partner falling in love and forming a deep attachment to another person (emotional infidelity). According to evolutionary theory, Buss *et al.* predicted that men would choose the sexual infidelity scenario while women would choose the emotional infidelity scenario. Results indicated that 49% of the 133 males selected the sexual infidelity scenario as more distressing, compared to only 19% of the women. A χ^2^ test of association indicated that the difference between the male and female proportions was statistically significant (*p* < 0.05).

Parametric and non-parametric observations can be analyzed using the Observation Oriented Modeling software. In this example, the observations (ordered as male/female and sexual/emotional infidelity) are clearly non-parametric, and an analysis similar to the χ^2^ test of association can be conducted. Employing a minimum degree of causal reasoning consistent with the evolutionary theory proposed by Buss *et al.*, a person’s gender (male/female) can be regarded as the cause of his or her choice between the two scenarios: *Gender –> Infidelity type choice*. The analysis in OOM proceeds by transforming the observations into a binary form referred to as their *deep structure* (see Grice [10], Chapter 2). Quantitative and non-quantitative observations can all be expressed in this same binary code, much like the information in a computer is stored in binary sequences. Once in this common form, the same analysis procedures, transformations, and randomization tests can be applied to the observations regardless of their parametric status. This greatly streamlines analyses and permits researchers to use a greater proportion of their intellectual resources on theory and model development rather than on choosing a particular statistical test. In this example, a binary Procrustes rotation was used to conform the effect (infidelity type choice) to the cause (gender). 

**Figure 1:**
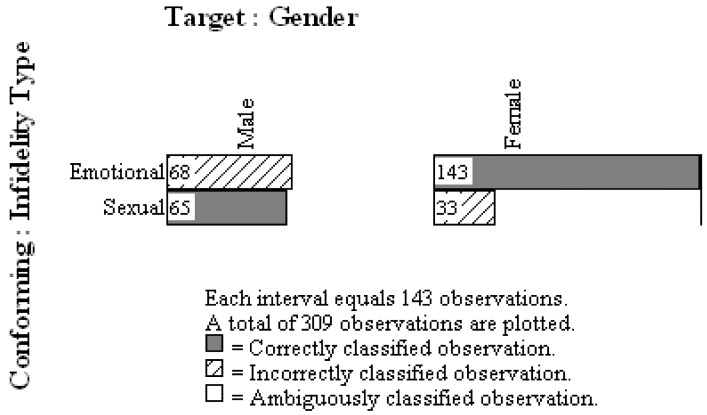
Multigram for *Gender –> Infidelity Type Choice* model.

In OOM visual examination of the observations, generally referred to as the “eye test”, is a meaningful and valuable evaluative technique. One of the primary graphing methods in OOM is the multi-level frequency histogram, or multigram. Figure 1 presents the multigram for Buss *et al.*’s data, and as can be seen, the distribution of ordered effect (conforming) observations are presented for each level, or unit, of ordered cause (target) observations. The observations are also presented as either correctly or incorrectly classified, and this determination is made on the basis of the overall pattern of results (see Grice [10], Chapter 4). In this instance, relative to males, more women chose the emotional infidelity as distressing, and these observations were therefore considered as “correct” classifications, as indicated by the dark grey bars in Figure 1. The males were more evenly split in their choices between the two infidelity types, but the sexual infidelity choices were considered as correctly classified when made by males. With regard to the “eye test” the proportions of sexual or emotional infidelity chosen by men and women appear starkly different. Indeed, the number of observations correctly classified in this instance is 208, or 67.31% of the original 309 participants. This percentage is referred to in OOM as the Percent Classification Correct (PCC) index, and it replaces all indices of effect size (e.g., *d*, *r*, *R*^2^, ω^2^) used in standard statistical analyses. Here, a moderate, but unimpressive, majority of the observations are correctly classified, suggesting that further theoretical work is needed to understand the causes presumably at work in the choice of infidelity type.

A randomization test is incorporated in OOM in order to aid the visual examination of the data and the PCC index. Specifically, the deep structures of the ordered observations are repeatedly shuffled and the same transformation (binary Procrustes rotation) conducted on each randomized version of the data. The investigator determines the number of randomized trials (e.g., 1000), and for each trial the PCC value is recorded by the OOM software. The PCC index from the actual observations is then compared to the generated distribution of randomized PCC values. The resulting probability statistic is referred to as a *c*-value (chance-value), rather than a *p*-value, in order to draw attention to the fact that no hypothetical distribution of scores is ever consulted in OOM. Moreover, the only assumption underlying the test is that the observations are completely independent. For the current data, the *c*-value is <0.0002, estimated from 5000 randomized versions of the observations. In other words, assuming the pattern in Figure 1 is a presentation of randomly ordered, independent observed units, less than one time in 5000 trials would a random shuffling of the deep structure of the ordered observations result in a PCC index of at least 67.31%. The *c*-value in OOM does not, therefore, provide a test of a null hypothesis in the tradition of null hypothesis significance testing which seeks to estimate population parameters (viz., a population proportions) from random samples of data. Instead, it is a means for evaluating whether or not the observations obtained via the research methods conform to a theory-consistent pattern with distinct regularity. The actual degree of conformity is provided by the PCC index and interpreted in the context of the multigram. The low *c*-value for the current data indicates that the observed pattern of observations is highly distinct, buttressing the obvious proportional gender differences in scenario choice.

**Figure 2:**
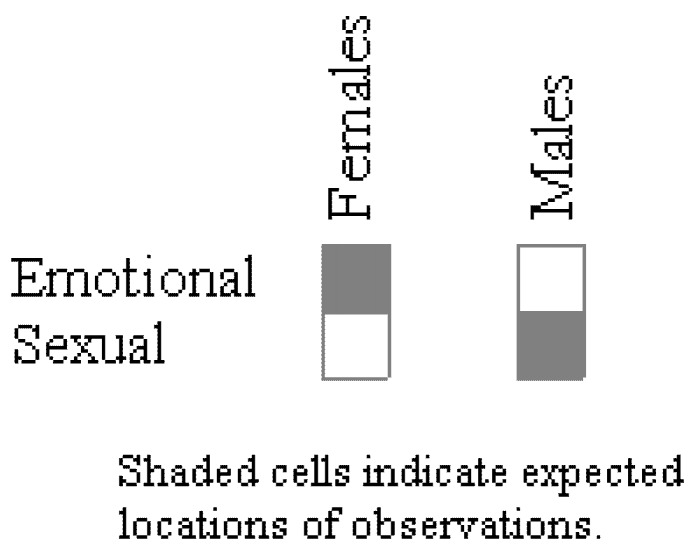
Expected pattern of observations according to evolutionary theory.

What is also plainly obvious in the pattern of results in Figure 1, however, is that the results for males in fact contradict the evolutionary hypothesis. The overwhelming majority of men, due to the evolutionary importance of avoiding being cuckolded, should have chosen sexual infidelity as distressing; however, only 49% of the men chose sexual infidelity. Buss *et al.* and subsequent researchers largely failed to see or to acknowledge the importance of this contrary evidence (e.g., [4,5,6,7,8,9,10,11,12,13,14,15,16]). From the standpoint of statistical analysis, this oversight was partly due to the fact that the χ^2^ test of association was not the appropriate analysis to conduct. Two, separate χ^2^ goodness-of-fit tests, one for males and one for females, should have been used instead. Equivalently, in OOM a simple pattern analysis tool can be used to determine if the data match expectation. With this tool the expected pattern of results is defined using the units of observation as shown in Figure 2, with males choosing sexual over emotional infidelity and females choosing emotional over sexual infidelity as more distressing. PCC indices and chance values are determined separately for males and females in the analysis. Results for the Buss *et al.* data reaffirm that, contrary to expectation, only 49% of the males chose sexual infidelity, and this difference was quite common in randomized versions of the observations (*c* ≤ 0.64, 5000 trials). By comparison, 81% of the females chose emotional infidelity as more distressing, and this high proportion was distinct (*c* < 0.0002, 5000 trials). 

It is instructive that while the original study by Buss *et al.* generated a great deal of interest and subsequent research by both detractors and supporters, the fact that the wrong statistical test was used to analyze the data was never seriously discussed. Even immediate detractors [15,16] appeared to accept the results and focused the brunt of their criticisms on the alleged causal mechanisms underlying the binary infidelity choice task. Cognitive theories were specifically put forth to offer alternative, causal explanations of the differences in proportions between the sexes. As will be discussed below, however, these efforts, as well as those of the evolutionary psychologists, were hindered by adherence to a script for conducting research that cannot yield the types of models necessary to truly understand causes and their effects. A necessary step toward building such *integrated models* is to shift from an attitude of estimating abstract population parameters for variables or for relationships between variables, as is done in null hypothesis significance testing, to an attitude of using graphical methods, the “eye test”, and chance values to examine observations for distinct patterns. The second example demonstrates this shift more clearly in the context of determining causality. 

## 3. Example 2: Causal Limitations of Variable-Based Models

**Figure 3:**
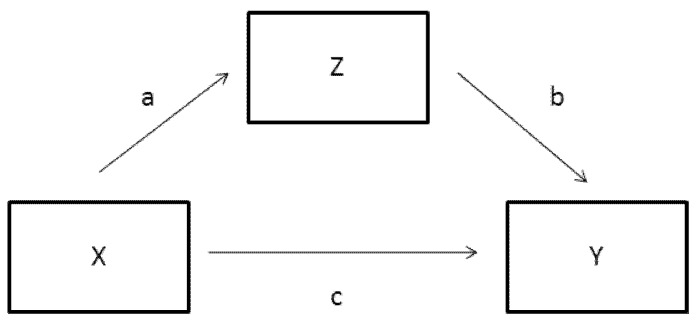
Standard mediation model showing both direct and indirect effects.

Recent years have witnessed a growing trend among quantitative psychologists and applied researchers toward attempting to infer causation from statistical methods. Drawing causal inferences from Structural Equation Modeling (SEM) has particularly been a topic of interest and debate among methodologists. Most notably, Pearl [17] has suffused the standard equations of SEM with particular assumptions and joined the equations to nodal graphing techniques in an attempt to create a causal inference engine. In a particular effort to derive a generic method for testing mediation models, Pearl [18] presents contrived data to demonstrate his approach. His example entails three dichotomous variables labeled X, Z, and Y. The first variable, X, represents a drug treatment (drug/no drug), the second variable, Z, stands for the presence of a certain enzyme in the blood stream (enzyme/no enzyme), and the third variable, Y, represents physical recovery from an ailment (cured/not cured). Drug treatment is the initial cause, the enzyme is the mediator, and recovery is the outcome. The three variables are linked in a standard mediation model format showing both direct and indirect connections between X and Y, as can be seen in Figure 3. 

The model is presumed valid in the derivation of Pearl’s Mediation Formula ([18], p. 27), and in the context of OOM it is referred to as a variable-based model. Variable-based models have been part of psychology’s Modal Research Practice for at least 70 years and are routinely represented in diagrams like Figure 3. In their simplest form they may link an independent variable to a dependent variable, and in their most complex form they may link numerous latent variables together in a presumed causal web. Three features are common to variable-based models. First, they are comprised of variables rather than explicit structures or processes such as those found in biochemical models or Bohr’s classic model of the atom. Second, the links between variables are routinely determined through analysis of statistical aggregates, such as means, variances, and covariances. With a more complex variable-based model, overall fit may in fact be determined by how well the observed variances and covariances can be reproduced by the estimated parameters within the model. Third, whether or not a link between two variables is considered worthy of inclusion in the model is determined most often through null hypothesis significance testing. It is because of these features that variable-based models are of limited value in the pursuit of the causes underlying observed phenomena. Pearl’s example, the results for which are presented as proportions in the Appendix, offers a clear case for demonstrating these limitations, especially when analyzed with OOM.

**Figure 4:**
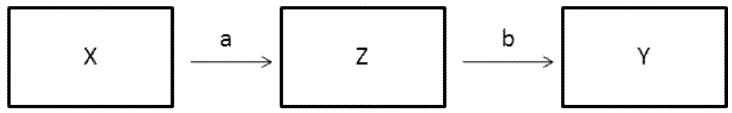
Variable-based model showing full mediation.

It is important to discriminate between what are referred to as mediation and moderation models in the variable-based literature. The model in Figure 3 is a mediation model, and if the direct link, *c*, is omitted, it becomes what is commonly referred to as a full mediation model, shown in Figure 4. Such models are correctly understood to reflect domino-like causes and effects [19]. In setting up and knocking down a line of dominos, force is applied to the first domino which falls into the second domino, which falls into the third, *etc.* After falling into the second domino, the first domino remains at rest and no longer serves as a direct cause of subsequent effects that are situated in time. For mediation models, then, time is necessary for understanding the cause and effect sequence. Considering the example data from the perspective of full mediation, the drug enters the blood stream and causes some organ or organs to secret the enzyme. In order to keep the example simple, let us assume the drug stimulates the liver to produce the enzyme. The enzyme then travels through the blood stream and eventually acts as the cause of the recovery. Again, to facilitate discussing the example, let us assume the enzyme neutralizes a virus, and this is the final effect (recovery or cure) in the sequence.

**Figure 5:**
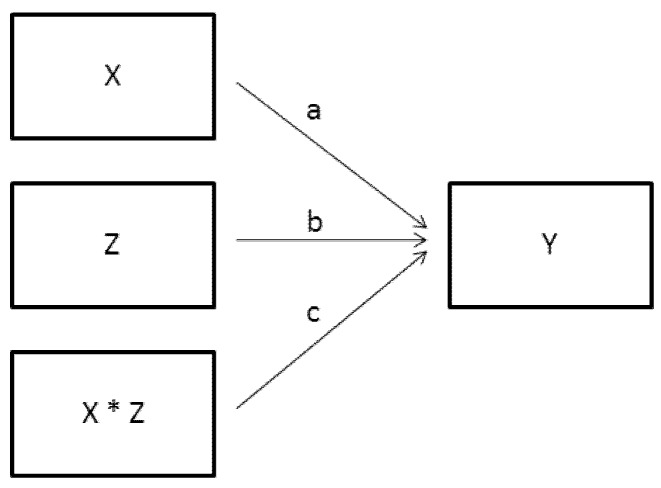
Standard variable-based moderation model.

A moderation model is entirely different, and is often represented graphically as shown in Figure 5. In standard regression terminology the product term (X * Z) represents an interaction between X and Z. More generically, it represents two causes that work together in some fashion to generate the effect. For the example data set a moderation model would include the drug and enzyme working together, simultaneously, to neutralize the virus. The drug, for instance, could enter the blood stream and chemically bond with the enzyme which is already present, creating a new substance with the capability of neutralizing the virus. With such a causal model it is not important to consider the drug as ordered in time before the enzyme. What matters is their “interactive” (read, chemically combined) effect on the virus. 

The full mediation and moderation figures above represent radically different models. Even with the example data set, with very few details regarding the variables and data collection available, two entirely different causal pathways leading to recovery from the virus can be discerned. In order to analyze the data as a full mediation model, a number of additional assumptions must first be made about how the observations were collected. It is not clear in Pearl’s [18] description of the example whether or not the enzyme can be present in the blood stream when the drug is administered. It is also unclear if the enzyme measurement is taken between the drug administration and diagnosis of recovery or at the time of diagnosis. Because the mediation model is assumed true, however, it seems reasonable to assume that the enzyme measurement was taken after the administration of the drug and before the final diagnosis. With full mediation it must also be assumed that the enzyme is not present in the blood stream at the beginning of the randomized experiment. Recall the example of the dominos. Imagine an experimenter setting up ten, separate ordered patterns of dominos. For half of the patterns the experimenter topples the first domino while for the other half the first domino is not toppled. In every case the second domino is in one state (erect) for both groups at the beginning of the experiment. Similarly, the enzyme is presumed absent in the drug example, thus yielding two ways in which a person’s observations (data) can match the cause-and-effect sequence as expected:

1. *Drug Administered –> Enzyme Secreted –> Virus Neutralized*
2. *Drug not Administered –> Enzyme not Secreted –> Virus not Neutralized*

In the context of OOM, observations that do not fit one of these two sequences indicate the operation of other causes or they indicate errors in making or recording the observations (e.g., mis-diagnosing the illness or mis-reporting the presence or absence of the enzyme). Naturally, the scientist must make every effort to reduce errors so that their impact may be regarded as trivial.

**Figure 6:**
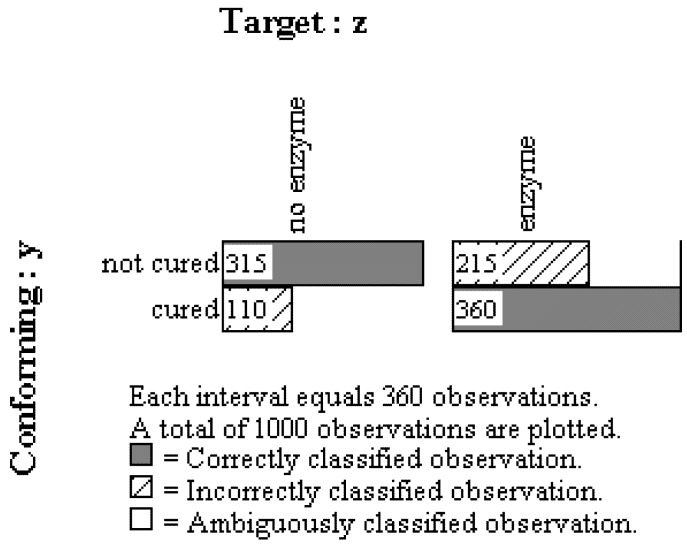
Multigram showing results conforming final effect (Y, cure) to mediating cause (Z, enzyme).

In order to test these sequences 1000 observations were generated based on the proportions reported by Pearl (see Appendix). As discussed in the evolution example above, observation oriented modeling revolves around the notion that effects should conform to their causes, which means that when analyzing full mediation one must work backward through the X –> Z –> Y sequence. The first step is thus to conform the last effect, Y (recovery), to its most proximate cause, Z (enzyme), the mediator. The results for this first step revealed that 67.50% of the persons could be classified correctly based upon the pattern of joint Z and Y observations. This PCC value was highly unusual as it exceeded every value obtained from randomized versions of the same observations (*c*-value < 0.0002, 5000 trials). As can be seen in Figure 6, consistent with expectation, most of the those cured (360/470 = 0.7660) secreted the enzyme while most of those who were not cured did not secrete the enzyme (315/530 = 0.5943). Contrary to expectation, however, 325 persons (32.50%) were not correctly classified and their observations were not consistent with the expectation that the enzyme caused the cure.

**Figure 7:**
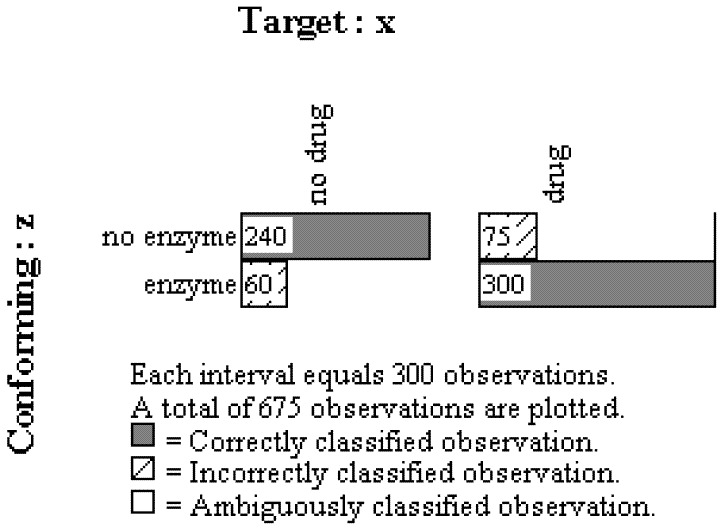
Multigram showing results conforming mediating cause (Z, enzyme) to initial cause (X, drug) for only those observations classified correctly in first step of the analysis.

The second step in the OOM analysis selects only those 675 individuals who were classified correctly in the first step. The mediator observations, Z (enzyme), are then brought into conformity with the original cause, X (drug treatment). Eighty-percent of the observations were classified correctly, and again this PCC value was highly unusual as indicated by the *c*-value from the randomization test (*c* < 0.0002, 5000 trials). The multigram in Figure 7, which includes only the 675 people who were correctly classified for the Z –> Y model above, reveals that the drug activated the liver to secrete the enzyme for most people (300/375 = 0.8000) while the enzyme was only found for a minority of those who did not ingest the drug (60/300 = 0.20). For those who did not ingest the drug, most did not produce the enzyme (240/300 = 0.8000). 

In summary, most of the individuals who consumed the drug were also found to possess the enzyme while most of those who did not ingest the drug were found to not have the enzyme. These 300 and 240 correctly classified persons matched expectations in terms of the causal link between the drug and enzyme. Also recall that the 675 people in this second step of the analysis were correctly classified in the first step of the analysis. Consequently, 54% of the observations, [((240 + 300)/1000) × 100], could be successfully traced through the X –> Z –> Y causal model.

**Figure 8:**
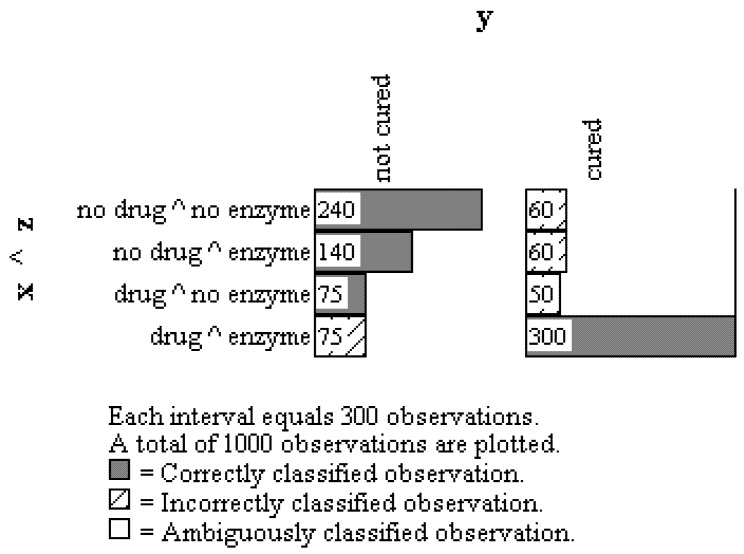
Multigram showing summary pattern for all observations on the three orderings: drug ingestion (X), enzyme secretion (Z), and disease state (Y).

A way to summarize these results in OOM and clarify the analysis is to cross the X and Z observations and present them in a multigram with the Y observations as shown in Figure 8. It should be noted this summary is similar to von Eye’s person-centered mediation modeling approach [20]. The multigram in Figure 8 shows the 240 and 300 people who could be successfully traced through the X –> Z –> Y causal sequence. It also shows the 460 people who violated the causal sequence in one way or another; for instance, 140 people did not ingest the drug but produced the enzyme and still were not cured, 75 people ingested the drug but did not produce the enzyme and were not cured, *etc.*

The overall conclusion is that a slight majority (54%) of the observations obtained from the 1000 persons in the study were patterned in a manner consistent with the full mediation model. There were numerous instances of individuals (n = 75) who consumed the drug, produced the enzyme, and still were not cured, as well as numerous instances of individuals (n = 60) who did not consume the drug nor produce the enzyme but were yet cured. Three-hundred twenty-five individuals violated the X –> Z –> Y sequence in other ways. Therefore, while these results are provocative they clearly reveal an inadequate understanding of the causes underlying the pattern of observations.

By comparison, Pearl’s [18] conclusion from the variable-based mediation analysis indicates enough confidence in the results to go forward with instituting policy changes:

“We conclude that 30.4% of those recovered owe their recovery to the capacity of the treatment to stimulate the secretion of the enzyme, while only 7% of recoveries would be sustained by enzyme stimulation alone. The enzyme seems to act more as a catalyst for the healing process of X than having a healing action of its own. The policy implication of such a study would be that efforts to substitute the drug with an alternative stimulant of the enzyme are not likely to be effective, the drug evidently has a direct effect on the disease, independent of, albeit enhanced by enzyme stimulation” (p. 28).

This summary does not match the conclusions drawn from OOM because it conflates the mediation and moderation models in Figure 4 and Figure 5 above. On the one hand, “30.4% of those recovered owe their recovery to the capacity of the treatment to stimulate the secretion of the enzyme.” This statement appears to support the X –> Z –> Y mediation sequence. On the other hand, the statement, “the enzyme seems to act more as a catalyst for the healing process of X than having a healing action on its own,” clearly violates the mediation model; instead, a moderated effect is suggested, such as the catalyst enzyme some way altering the chemical composition of the drug, making it more effective in destroying the virus. This moderation interpretation would require a more sophisticated model that would include structural changes in the drug *after* it has stimulated the production of the very enzyme that will change it. Moreover, aspects of Pearl’s conclusions do not pass the “eye test.” Most notably, the claim that “...the drug evidently has a direct effect on the disease...” is contradicted by the multigram in Figure 8 which clearly shows that of the 125 persons who consumed the drug but *did not* produce the enzyme, 75, a majority (60%), were *not* cured. It is therefore difficult to examine the pattern of observations and justify the drug acting directly and alone to kill the virus and cure the disease. 

The confounding of mediation and moderation effects is avoided in OOM which is a step in the right direction toward theoretical clarity and toward understanding causal mechanisms. It is important to emphasize, however, that while the OOM analyses above were straightforward, they required more information regarding the observations than the variable-based approach. For instance, since the information was not provided for the contrived example, it was assumed that the enzyme was not present in the blood streams of the participants at the beginning of the clinical trials. Two-hundred people, however, did not consume the drug and yet the enzyme was observed. With genuine research this anomaly would prove critical, and such information would be available and taken into account accordingly. The fact that critical information is not required when using variable-based models points to their inadequacy to represent fully the structures and processes that constitute phenomena. For this example the researcher is never challenged to search for the causes through careful study of the biochemical structures of the drug, enzyme, and virus and the processes that bind them together. In psychology variable-based models may be useful for revealing aggregate associations among sets of variables, but they are equally insufficient for representing the complex causes of psychological phenomena and human behavior. More flexible and complex, integrated models are necessary.

## 4. Example 3: Toward Integrated Models

**Figure 9:**
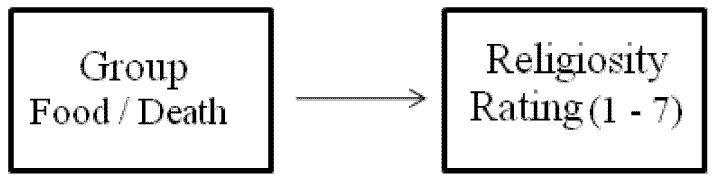
Simple variable-based model for study of Terror Management Theory.

The move from variable-based modeling to observation oriented modeling finally hinges on a researcher’s capability of sketching an integrated model. In the early stages of studying a given natural phenomenon such a sketch will likely be crude, but even a crudely sketched model can direct research in a systematic and more fruitful way compared to the current Modal Research Practice (MRP) in psychology. As an example, consider a simple study of Terror Management Theory by Norenzayan and Hansen [21] in which undergraduate students were randomly assigned to two conditions. In the experimental condition, the students wrote for approximately five minutes about what they thought would happen to them when they die. In the control condition, the students wrote about their favorite foods for approximately five minutes. All students then completed a simple short-term memory task designed to clear their working memories of the material they wrote in their essays. Finally, students conducted ratings using a 7-point Likert-type scale in response to the following prompt: “How religious are you?” Based on Terror Management Theory and using MRP terminology, the study authors expected statistically significant differences between the death and food groups such that the population mean for the former would be estimated to be higher than the population mean for the latter group. The independent variable in the study, which is presumed to be the cause, is group membership, and the dependent variable is the religiosity question, which is presumed to be the effect. The variable-based model can be represented simply, as shown in Figure 9, and the results from an independent samples *t*-test revealed a significant association between the two variables (*p* = 0.05). It is noteworthy that Norenzayan and Hansen reported three additional studies in the same manuscript, not one of which was an exact replication of this first study.

Going beyond the simple variable-based model and the MRP requires the development of an integrated model. Such a model should attempt to represent the structures and process constituting the natural phenomena under investigation; that is, it should provide a sketch of the causes and their effects. From the standpoint of moderate realism causes are understood from a fourfold perspective: material, formal, efficient, and final cause. Material cause refers to the matter of which a thing is composed, formal cause refers to the shape or form of the thing that plays a key role in its intelligibility, efficient cause refers to changes in time that bring about a thing or given state, and final cause refers to the purpose for which an action is performed or to the natural end state of a given process. Wallace [22] provides an accessible treatment of Aristotle’s four causes, and Grice [10] provides examples incorporating the four causes into psychological theories. 

**Figure 10:**
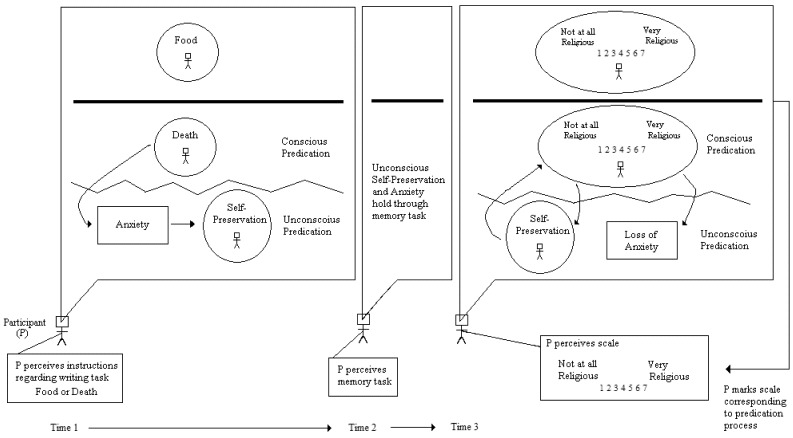
Integrated model for study of Terror Management Theory.

Terror Management Theory is generally understood as an efficient cause model, where a psychological state (anxiety) causes different behaviors (e.g., believing in God). Figure 10 shows an integrated model that attempts to explicate this causal understanding of Norenzayan and Hansen’s study. As can be seen, the focal point of the model is the human participant and the events which take place in his or her psyche as well as in the environment. There is an initial lesson already to be learned here from the integrated model. It is *only at the level of the person* that causes are understood to operate in the model, *not at the level of a computed aggregate* such as a mean, median, or mode (see Harré [23]). Most theories in psychology, like Terror Management Theory, do not explicitly make predictions about aggregate statistics. As Skinner [8] understood, this logical inconsistency points to serious, limiting aspects of large-sample, between-subject research designs and the MRP. By placing the focus on the units of observation and patterns of observations, observation oriented modeling attempts to overcome these limitations.

The integrated model in Figure 10 tracks the person through three time points: the writing part of the study, the memory test, and the final rating. The events that take place within the person’s psyche are represented in the callouts, and the two conditions in the study are separated by the bold line in each. The control (food) condition is located above the line while the experimental (death) condition is represented below the line. A circle or ellipse in a callout represents the predication process whereby a person makes an attribution or judgment about himself or herself or about some aspect of the study. For instance, in the experimental condition the student is asked to apply the predicate “death” to oneself by writing about what will happen when death occurs (which is a certainty in the future). A circle enclosing a stick figure representing the participant and the word “death” represents this process. How does this predication process actually occur? The model does not make this explicit, and this is the second lesson to learn from an integrated model. An integrated model provides an explicit framework for other scholars to ask specific questions about its structures and processes and about how to expand upon the model. Often such expansions will involve greater detail that represents an ever-increasing understanding of nature’s complexity. The situation is analogous to the development of the atomic model, which began as a simple model that was analogous to our solar system (viz., electrons following orbits around a core of neutrons and protons), and which is now much more complex. While most research in Terror Management Theory has been undertaken by social and personality psychologists, the integrated model also provides a framework for integrating different domains of research. A cognitive psychologist, for instance, could expand the predication icons to explain exactly how persons make attributions about themselves.

Once the person applies the predicate “death” to himself or herself, the model in Figure 10 posits that this predication causes (efficient cause) an unconscious state of anxiety that in turns activates (again, efficient cause) an unconscious predication to preserve oneself in existence. At this point devotees of Terror Management Theory may argue that the anxiety is not yet unconscious. It is only during the second stage of the experiment, the memory task, that anxiety drops from awareness. Disagreement may also be voiced against the self-preservation predication. Is it even necessary? This is the third lesson to learn from the integrated model. The author of the model must make the details of the causes and their effects explicit. By doing so disagreements will likely lead to much more fruitful dialogue because the vagaries inherent in simple variable-based models are avoided. The level of theoretical reasoning required to build and defend the model in Figure 10 is vastly greater than what is required to draw two boxes connected by an arrow (Figure 9). A well-reasoned and more detailed model will generate more specific questions which will in turn lead to more thorough and convincing tests of the model’s components. For instance, if anxiety is indeed posited to be outside of awareness during the first phase of the study, then methods must be employed to obtain the necessary observations to verify this expectation.

In the second phase of the study (Time 2 in Figure 10), the student works consciously on the memory task while the unconscious self-preservation predication is hypothesized to remain active. Finally, at the third time point in the study the student is asked to rate himself or herself on the 7-point Likert-type scale in response to the “How religious are you?” prompt. Here a fourth lesson is learned from the integrated model. Most psychologists use such rating scales because they assume the attributes under investigation are structured as continuous quantities. In this example the 7-point scale serves as a crude representation of the religiosity attribute, but is religiosity structured as a continuous quantity? The question is never asked by the study authors nor is any effort made to even justify the particular scale. Would a 9-point scale yield more meaningful responses? Would a 5-point scale be adequate, or perhaps an analog scale anchored by “not religious” and “religious?” Most contemporary measurement specialists might even argue that multiple items instead be employed and combined into a “latent variable” on which the average responses could be compared between groups. Such an approach would still assume that religiosity is structured as a continuous quantity. From the perspective of observation oriented modeling, however, continuous quantitative structure is not an assumption; rather, as convincingly argued by Michell [24,25], it is a scientific hypothesis that must be tested. Absent a demonstration of continuous quantitative structure, the observations obtained from any measurement procedure must be considered as discrete or as judgments of order. With observation oriented modeling, then, the measurement issue must be addressed head-on rather than relegated to an assumption within a variable-based model. In no way, however, does this lesson imply that all, or even any, psychological attributes are structured as continuous quantities. The point is that the psychologist must make every effort to insure that attributes are represented faithfully in the integrated model with regard to their natures. For instance, a psychologist developing an integrated model based on George Kelly’s [26] Personal Construct Theory would represent every act of predication in the form of a bipolar structure (*i.e.*, a personal construct). Religiosity has not been demonstrated to possess continuous quantitative structure, and therefore the use of the 7-point Likert-type scale must be questioned. 

**Figure 11:**
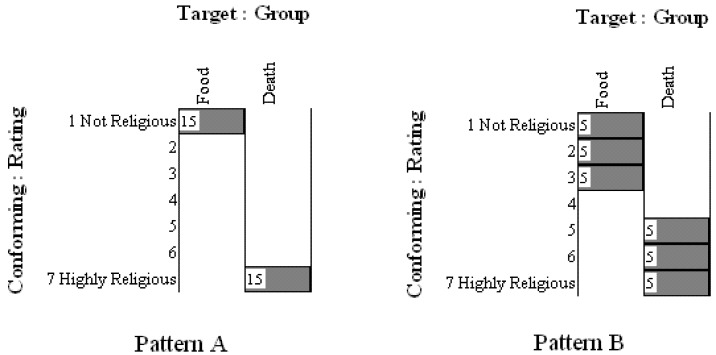
Hypothesized patterns of observations for study of Terror Managemnt Theory

Without the assumption of continuous quantitative structure, the use of aggregate statistics also becomes secondary. Instead, the goal is to focus on how the observations are ordered (e.g., as 7 units or 5 units from a rating scale) and on the patterns of observations. The integrated model in Figure 10 shows the person using the 7-point scale in either the control or experimental conditions. Based on the integrated model and how the observations are ordered via the scale, what pattern of results would confirm the model? Figure 11 shows two of many possible multigram outcomes for 30 hypothesized participants. As can be seen in Pattern A, all 15 persons in the control condition rated themselves “1” (not religious) on the Likert-type scale while all 15 persons in the experimental (death) condition rated themselves “7”. In the second hypothesized set of results (Pattern B) the persons in the food condition rated themselves from 1 to 3 while persons in the death condition rated themselves from 5 to 7. Would either or both of these patterns of results support the integrated model? Perhaps a different pattern is expected? Perhaps at this point it now becomes clear that the observations should be ordered into dichotomous units rather than 7 units? These questions demonstrate that observation oriented modeling requires a radically different way of thinking about psychological data. Rather than assuming continuous quantitative structure and estimating an abstract population parameter, such as a population mean using null hypothesis significance testing, the researcher is required to think clearly about how the observations are to be ordered into units and about what pattern of observations would support the accuracy of the integrated model.

**Figure 12:**
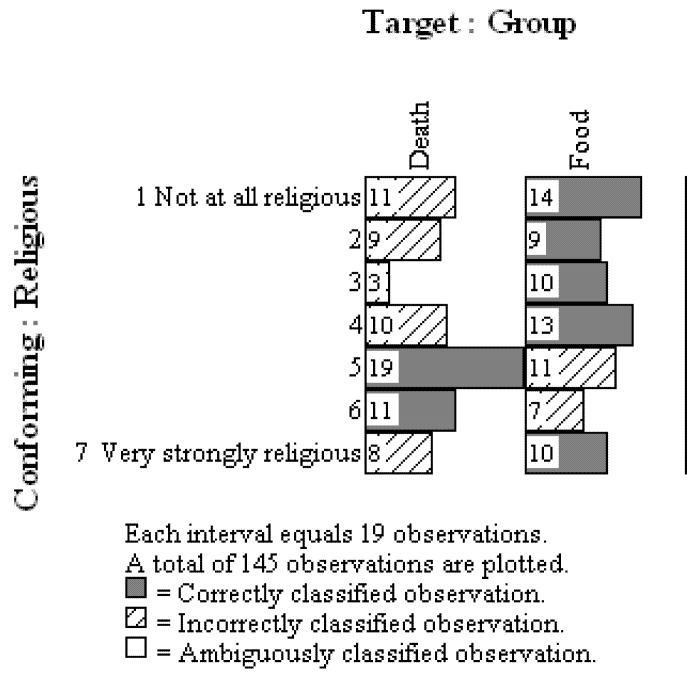
Multigram of actual observations for Terror Management Theory study.

For the purpose of working with genuine data to demonstrate the examination of patterns in the OOM software, we replicated Norenzayan and Hansen’s study as part of a course exercise at Oklahoma State University. Students were blind to the design and purpose of the study prior to participating, and they later analyzed their anonymous ratings using standard statistical methods (viz., descriptive statistics, independent samples *t*-test, and null hypothesis significance testing) and the OOM software. Over four semesters 145 students participated in the study. The observations were collected into one data set and the causal model, *Condition –> Religiosity Rating*, was tested. In other words, the effect was rotated to conformity with the cause, and results indicated that only 59.31% (86/145) of the observations could be classified correctly. This result did not exceed randomized versions of the same observations very often (*c* = 0.33, 5000 trials), indicating that the pattern of results was not unique. The multigram in Figure 12 shows clearly that the two sets of ordered observations for the control (food) and experimental (death) groups could not be discriminated. For each rating unit (1 to 7) the two groups were nearly evenly split, and no clear separation between observations as seen in the patterns in Figure 11 was achieved. The pattern of results thus failed to support the integrated model and Norenzayan and Hansen’s findings. This exercise therefore also demonstrates that even in the context of an integrated model, exact replication is still a hallmark of scientific investigation.

A final lesson to be learned from the integrated model in Figure 10 is the importance of final cause in theories of psychology. Humans are understood to engage in particular behaviors or in long series of behaviors over time according to the purposes they construct in their conscious minds. Such an idea is critical, for instance, for goal research in which the posited goals are considered as the final causes of behavior. Final cause can also be considered as the natural end states that processes achieve or that systems tend toward under normal circumstances. A rose bush, for instance, will grow to a height according to its genetic structure and environmental conditions. The process of growth will of course not continue forever, and at some point an observer will determine that the bush has reached its maximum height (an end state). The plant will continue to absorb nutrients, grow new leaves and stems, and react to any damage to its structure, but it will not grow any taller. Similarly, mixing two chemicals may result in a reaction in which the two combine, thus reaching a natural end state; and unless disturbed, the chemicals may stay bonded indefinitely. Terror Management Theory appears to be more consistent with this latter view of final cause as a natural end state, or homeostatic balance, than as conscious purpose. By writing about death, the person is disturbed from his or her normal psychological/emotional state; and by stating belief in God, the person returns to this normal state. The integrated model may therefore need to be significantly altered to take the form of a cybernetic model. Powers [27,28] has shown how such models can be constructed for psychological phenomena, and it is important to realize that such models are not necessarily deterministic. They simply allow the researcher to posit more complex, dynamic systems that may pave the way for modeling final causes as well as efficient and formal causes. More generally, Rychlak [29,30] argued thirty years ago for the necessity of adopting a richer view of causation in psychology, and integrated models may provide a pragmatic means for finally doing so. 

## 5. Conclusions

In the wake of Bem’s [1] published and highly publicized studies on psi phenomena, psychologists have again begun to critically evaluate their standard research practices and data analysis methods. These practices and methods took hold in psychology over 70 years ago with the final ascendancy of the Galtonian school of thought. Sound criticisms of psychology’s Modal Research Practice (MRP, [2]) can be traced back to those early years as well, and only time will tell if today’s criticisms have any lasting impact. From our standpoint, change will not occur unless psychologists begin to think differently about how they approach the human psyche and human behavior. As entailed in the three examples above, something of a Gestalt shift is needed in which psychologists abandon the positivism inherent in their MRP and variable-based models and adopt the philosophical realism underlying integrated models [31]. In a practical way, such a shift involves a step toward one’s data. Senior psychologists have been known to lament the day when sophisticated statistical analyses performed on large data sets on a powerful computer replaced clear thinking and a personal familiarity with one’s data. Observation oriented modeling (OOM) attempts to re-unite researchers with their data through simplified analysis routines and an emphasis on visual examination of original observations. By comparison null hypothesis significance testing is a mathematical/statistical scheme that is highly abstract, which partly explains why studies have shown that most psychologists cannot properly define the *p*-value...the very thing they rely upon to determine if their results are worth pursuing further or publishing [32]. The first example above involving gender differences in jealousy demonstrated the common-sense aspects of OOM. Rather than estimating abstract population parameters, the goal was to visually examine the patterns of original observations in order to judge whether or not they supported evolutionary theory. Opposite to null hypothesis significance testing, the probability value incorporated in OOM (the *c*-value) was of secondary interest, provided only to help prevent the researcher from reading too much into a pattern of results that may appear overly distinctive. The primary numerical outcome from the OOM software that accompanied the multigram was the Percent Correct Classification (PCC) index which indicated the number of observations correctly classified by the analysis. Raw accuracy, not a probability statistic, is therefore the criterion for judging one’s results and consequently one’s understanding of the natural phenomena under investigation. 

The second and third examples showed that the PCC index replaces measures of effect size employed in variable-based models, even though most of these latter measures (e.g., *d*, *r*, *R*^2^, ω^2^) are based on aggregate statistics. Because OOM works at the common level of deep structure for all observations, the PCC index can be computed for quantitative and non-quantitative observations. Its centrality in the observation oriented modeling approach also fits well with the American Psychological Association’s recent call for reforms in data analysis techniques that include a shift in emphasis from null hypothesis significance testing to the reporting and interpretation of effect sizes [33]. The second example furthermore demonstrates, however, that the PCC index must not be treated as an index of “effect size” in the standard sense because it should always be accompanied by at least a crude causal model that seeks to explain the pattern of obtained observations. The goal of the analyses in the drug/enzyme/cure example was to trace observations through a full mediation model, which represented a domino-like chain of causes and effects. The variable-based approach to that particular example resulted in a number of aggregate statistics that appeared to confound two distinct causal models of mediation and moderation. Moreover, with the variable-based analysis troubling patterns in the data clearly present to the “eye test” were missed; for instance, the variable-based results were interpreted to support a direct effect of the drug curing the disease, but a multigram revealed that most individuals who took the drug and failed to secrete the enzyme were not cured (*i.e.*, cured by the drug alone). The OOM analyses avoided these pitfalls and required an effort be made to begin laying down the specific causal pathways between the drug, enzyme, and cured disease. Because of the commitment to a full mediation model, missing details of how the data were collected (e.g., was the enzyme present in the blood stream at the beginning of the trials?) became important. If the example represented a genuine problem, then a great deal more information would be needed to understand exactly how the drug causes a cure. Indeed, the scientist would eventually need to work at the level of biochemical structures and processes to arrive at a truly causal understanding of how the drug, enzyme, and disease are intertwined. OOM challenges researchers to develop such detailed models which go far beyond connecting nodes or boxes with lines in a variable-based model. 

The third and final example indeed provided a crude integrated model for a study on Terror Management Theory. Again, this model attempted to represent the structures and processes constituting the phenomena under investigation in the context of the particular study. It was much more challenging to develop than the simple variable-based model connecting the independent and dependent variables, and it is interesting to imagine how Norenzayan and Hansen’s [21] research might have unfolded if they had attempted to develop such a model. Instead of attempting to test a generic connection between thoughts of death and religious beliefs, as they did with their four studies, they may have instead taken the first steps toward developing a truly causal understanding of anxiety, knowledge of death, and religious beliefs. Of course, they would have also first attempted to replicate their initial study. The failed replication reported above suggests their initial results led them down the wrong path and that they need to retrace their steps to correct the error. It is also interesting to imagine how different the history of Buss *et al.*’s (first example above, [13]) controversial research would have been if they had attempted to develop an integrated model explaining exactly how reading the scenes of infidelity caused particular ratings for men and women. An even greater challenge would be to sketch an integrated model describing exactly how the alleged sex differences emerged over time in the evolutionary history of humans. 

In any case, by employing Aristotle’s four causes, integrated models are likely to prove more capable of explaining the human psyche or human behavior than variable-based models because they are more amenable to thinking about systems. When we think about persons as systems rather than as collections of variables to be measured and correlated, then a richer and more dynamic picture emerges. Understanding any system will require knowledge of the material aspects of the system and how that material is organized (material and formal cause; e.g., genetic information) as well as time-dependent processes that occur within the system and that impact the system from the outside (efficient cause; e.g., a threatening image that leads to an increase in heart rate and a heightened state of arousal). All systems are also understood to have normal resting states (final cause; e.g., normal body temperature), and persons are further understood to strive for particular end states or goals (final cause). With observation oriented modeling, then, the intellectual move is one from a variable-based view of nature to a systems-based view of nature (see [28]). Such a change in viewpoint, accompanied by innovated methods of data collection and analysis, may finally light the way for a more productive future in psychological research. 

## Appendix

Beginning with Table 5 on page 28 of Pearl’s [18] manuscript, 1000 cases were generated, 500 of whom received the drug treatment and 500 of whom did not. Of the first group, 200 produced the enzyme. Of the second group, 375 produced the enzyme. What follows is a frequency histogram from the OOM software with 1000 observations consistent with the proportions reported in Table 5:

The “40%” and “75%” labels and computations have been added to highlight the values reported in Table 5. Next, values for the third Y variable were generated to match the proportions in Pearl’s Table 4. The histogram with the proportions are as follows:

## References

[1] Bem D.J. (2011). Feeling the future: Experimental evidence for anomalous retroactive influences on cognition and affect. J. Pers. Soc. Psychol..

[2] Lebel E., Peters K. (2011). Fearing the future of empirical psychology: Bem’s (2011) evidence of psi as a case study of deficiencies in modal research practice. Rev. Gen. Psychol..

[3] Roberts B.W. (2011). Personality psychology has a serious problem (and so do many other areas of psychology). P: The Online Newsletter for Personality Science.

[4] Alcock J. (2011). Back from the future: Parapsychology and the Bem affair. Skeptical Inquirer.

[5] Bakan D. (1967). On Method: Toward a Reconstruction of Psychological Investigation.

[6] Meehl P.E. (1978). Theoretical risks and tabular asterisks: Sir Karl, Sir Ronald, and the slow progress of soft psychology. J. Consult. Clin. Psych..

[7] Danziger K. (1990). Constructing the Subject.

[8] Skinner B.F. (1956). A case history in scientific method. Am. Psychol..

[9] Wagenmakers E.J., Wetzels R., Borsboom D., van der Maas H. (2011). Why psychologists must change the way they analyze their data: The case of psi. J. Pers. Soc. Psychol..

[10] Grice J.W. (2011). Observation Oriented Modeling: Analysis of Cause in the Behavioral Sciences.

[11] Pearson K. (1892). The Grammar of Science.

[12] Lamiell J.T. (2011). Statisticism in personality psychologists’ use of trait constructs: What is it? How was it contracted? Is there a cure?. New Ideas Psychol..

[13] Buss D., Larsen R., Westen D., Semmelroth J. (1992). Sex differences in jealousy: Evolution, physiology, and psychology. Psychol. Sci..

[14] Buss D.M., Larsen R.J., Westen D. (1996). Sex differences in jealousy: Not gone, not forgotten, and not explained by alternative hypotheses. Psychol. Sci..

[15] DeSteno D.A., Salovey P. (1996). Evolutionary origins of sex differences in jealousy? Questioning the “fitness” of the model. Psychol. Sci..

[16] Harris C.R., Christenfeld N. (1996). Gender, jealousy, and reason. Psychol. Sci..

[17] Pearl J. (2009). Causality: Models, Reasoning, and Inference.

[18] Pearl J., Hoyle R.H. (2012). The causal foundations of structural equation modeling. Handbook of Structural Equation Modeling.

[19] Collins L., Graham J., Flaherty B. (1998). An alternative framework for defining mediation. Multivar. Behav. Res..

[20] Von Eye A. (2002). Configural Frequency Analysis: Methods, Models, and Applications.

[21] Norenzayan A., Hansen I. (2006). Belief in supernatural agents in the face of death. Pers. Soc. Psychol. B.

[22] Wallace W.A. (1996). The Modeling of Nature.

[23] Harré R., Reason P., Rowan J. (1981). The positivist-empiricist approach and its alternative. Human Inquiry.

[24] Michell J. (1999). Measurement in Psychology: Critical History of a Methodological Concept.

[25] Michell J. (2011). Qualitative research meets the ghost of Pythagoras. Theor. Psychol..

[26] Kelly G.A. (1955). Behavior: The Control of Perception.

[27] Powers W.T. (2005). Behavior: The Control of Perception.

[28] Powers W.T. (2008). Living Control Systems III: The Fact of Control.

[29] Rychlak J.F. (1985). A Philosophy of Science for Personality Theory.

[30] Rychlak J.F. (1988). The Psychology of Rigorous Humanism.

[B31-behavsci-02-00001] 31.Moving from positivsm to realism here primarily entails revitalizing three important aspects of an Aristotelian view of science. First, science must be regarded as the investigation of causes and effects rather than as mere description and prediction. Second, the four-fold (material, formal, efficient, final) view of causality must thoroughly supplant Hume's skeptical notion of causation. Finally, if causes are understood as inhering in the things of nature, then any pursuit of causes must assume that the things of nature are indeed knowable, contrary to Kant's famous dictum. An ancillary concern regards overturning the "quantitative imperative" [24,25] that assumes the psychological phenomena worth studying are structured as continuous quantities...a bias rooted in Cartesian philosophy.

[32] Gigerenzer G. (2004). Mindless statistics. J. Socio-Econ..

[33] Wilkinson L. (1999). Task force on Statistical Inference. Statistical methods in psychology journals: Guidelines and explanations. Am. Psychol..

